# Predictors of acute cardiovascular events following acute exacerbation period for patients with COPD: a nested case–control study

**DOI:** 10.1186/s12872-020-01803-8

**Published:** 2020-12-10

**Authors:** Wei-ping Hu, Tsokyi Lhamo, Feng-ying Zhang, Jing-qing Hang, Yi-hui Zuo, Jian-lan Hua, Shan-qun Li, Jing Zhang

**Affiliations:** 1 Department of Pulmonary and Critical Care Medicine, Zhongshan Hospital, Shanghai Medical College, Fudan University, Shanghai, 200032 China; 2 Department of Respiratory Medicine, Tibet Autonomous Region People’s Hospital, Tibet, China; 3 Department of Respiratory Medicine, Putuo District People’s Hospital, Shanghai, China

**Keywords:** Acute exacerbation of chronic obstructive pulmonary disease, Acute cardiovascular events, Heart rate, Electrolyte disturbance, Diuretics

## Abstract

**Background:**

It has been noted that there is an increase in the incidence of acute cardiovascular events (CVEs) in patients with chronic obstructive pulmonary disease (COPD) during an acute exacerbation (AE), thereby causing increased inpatient mortality. Thus, we have tried to identify predictors of acute CVEs in patients with AECOPD via a nested case–control study.

**Methods:**

A total of 496 cases hospitalized for AECOPD were included in this study, and followed-up for up to 6 months after discharge. Acute CVEs in the AE period were defined as a new or worsening acute coronary syndrome (ACS), arrhythmia, or left ventricular disfunction (LVD). Predictors of CVEs were selected from several variables, including baseline characteristics and treatments in the stable period as well as symptoms, laboratory tests, complications and treatments in the AE period.

**Results:**

Thirty cases (6.05%) had acute CVEs, namely 2 had ACS, 13 had LVD and 19 experienced some form of arrhythmia. Four deaths were observed in the CVE group, with significantly increased death risk compared with the non-CVE group (*P* = 0.001, OR = 5.81). Moreover, patients who had CVEs were more prone to have re-exacerbation within 3 months. Multivariate analysis showed that previous LVD history (*P* = 0.004, OR = 5.06), 20% increase in heart rate (HR) (*P* = 0.003, OR = 10.19), electrolyte disturbance (*P* = 0.01, OR = 4.24) and diuretics (*P* = 0.002, OR = 6.37) were independent predictors of CVEs. In addition, usage of theophylline, fluoroquinolone and inhaled beta agonists in the AE period were not statistically associated with acute CVEs.

**Conclusions:**

Our preliminary study indicates that patients hospitalized for AECOPD with previous LVD history or increased HR need close observation and diuretics should be cautiously used with regular electrolyte monitoring. These findings need to be confirmed in a large cohort.

## Introduction

Chronic obstructive pulmonary disease (COPD) is a chronic lung disease characterized by irreversible airflow limitation and progressive decline of lung function, and it is the third leading cause of death in the world [1, 2]. Several cohort studies reported that cardiovascular disease (CVD) is one of the top three leading causes of death in patients with COPD, following respiratory infections and respiratory failure [3–5]. Moreover, previous history of CVD can additionally increase risks of death in elderly COPD patients with pneumonia [6].

Patients with COPD tend to have concurrent CVDs with a prevalence of 28–70% [7]. Hyperlipidemia (45%) and hypertension (43%) have been found to be the most common cardiovascular risk factors [8]. Increased risks of acute cardiovascular events (CVEs) namely acute coronary syndrome (ACS) [9], arrhythmias [7] and sudden cardiac death [10] have also been reported in the patients with COPD. A large cohort study demonstrated that the prevalence of heart failure among patients with COPD is significantly increased, leading to a higher all-cause mortality [11]. During a period of acute exacerbation (AE), aggravated hypoxia and systemic inflammation are precipitating factors for acute CVEs, especially in the high-risk population [10, 12, 13]. Therefore, clinicians should remain vigilant for early acute CVEs following AECOPD and identify risk factors, especially the use of potentially-inappropriate medication. However, knowledge of risk factors of CVEs in patients with AECOPD is still incomplete.

Traditional risk factors of CVDs include previous history, hypertension, diabetes, hyperlipidemia and hyperuricemia. In addition, serious side effects of certain drugs might be the chief culprits of some CVE cases. A case–control study reported that theophylline could increase the risk of arrhythmias and acute heart failure by 80% [14]. Moreover, a meta-analysis showed that fluoroquinolones increased the risk of arrhythmias and cardiovascular death by 80% and 71% [15]. The relationship between inhaled beta-receptor agonists (bronchodilators) and CVDs is still highly debated, for beta-receptor antagonist is well-known therapy of cardiac remodeling and heart failure [14]. In the patients with COPD, initiation of inhaled bronchodilators has been related to short-term elevated risks of severe CVEs [16], and adding a second bronchodilator to the previous monotherapy also slightly increased the risk of heart failure after a one year period [17]. On the other hand, dual bronchodilators (beta-receptor agonists and muscarinic receptor antagonist) have been shown to improve left ventricular filling by reducing lung hyperinflation [18].

Herein, we performed a nested case–control study in a prospective COPD cohort in Shanghai, with the aim of screening the predictive factors of acute CVEs following the onset of AECOPD.

## Method

### Patient recruitment and data collection

We performed a nested case–control study within a prospective cohort. Between January 2015 and July 2017, we recruited patients hospitalized for AECOPD in the Department of Pulmonary Medicine of Shanghai Zhongshan Hospital and Shanghai Putuo District People’s Hospital into our prospective cohort.

At the time of admission, patients with a clearly recorded COPD history were interviewed by two separate pulmonologists, to evaluate whether their deteriorated respiratory symptoms were categorized as AECOPD. Exclusion criteria were exacerbations induced by other respiratory diseases, including asthma, bronchiectasis, congestive heart failure, pulmonary embolism, pleuritis, restrictive lung disease and pneumothorax.

Baseline information and conditions during the stable period were recorded at admission; these consisted of demographic characteristics, COPD-associated evaluation (risk factors, lung function, assessment scales and number of previous AE), COPD-associated treatments during the stable period (inhaled agents, oral drugs, assistive breathing and vaccination) and comorbidities (common CVDs, other respiratory diseases and other common diseases). Modified Medical Research Council (mMRC) dyspnea scale [19] and COPD assessment test (CAT) [20] were used to stratify the severity of dyspnea and measure COPD’s adverse effects on daily life.

Individualized treatment for each patient in the hospitalization period was carried out according to personal conditions and documents of *Global Strategy for Prevention, Diagnosis and Management of COPD* (GOLD). Detailed examination and therapeutics in the AE period were recorded, including new or worsening manifestations, vital signs at admission, laboratory tests (blood routine examination, blood biochemistry, coagulation tests, arterial blood gas analysis, etc.), sputum culture, computed tomography, assistive breathing and drug usage (antibiotics, inhaled bronchodilators, inhaled or systemic steroids and others). In addition, we also collected information about emerging or worsened complications (pneumonia, PE, pneumothorax, acute coronary syndrome [ACS], arrhythmia, left ventricular dysfunction [LVD] and others).

At the timepoint of 1, 3 and 6 month after discharge, patients were followed up either by outpatient department visits or by telephone, to prospectively collect information about any recurrent acute exacerbation (re-AE) and survival.

Electrolyte disturbance was defined as abnormal electrolyte (including sodium, potassium, calcium, calcium, phosphorus and magnesium) value(s) which were not within the normal range during the blood tests carried out on admission. Diuretics used included torasemide, spironolactone and furosemide. We also noted the category and doses of diuretics prescribed as well as their initiation dates. Methylxanthines used included aminophylline, doxophylline and diprophylline.

### Outcome

Primary outcome was any CVEs, including emerging or worsening ACS, LVD and arrhythmia occurring during the AE period. The clinical notes of cases with CVE were retrieved and carefully reviewed to determine the chronological sequence of the use of diuretics, appearance of electrolyte disturbance and CVEs. Secondary outcomes were defined as days of stay in hospital, hospitalized death, death within1, 3 and 6 months, and recurrent AE within1, 3 and 6 months. Re-AE was defined as the new worsening of respiratory symptoms lasting for over 2 days, which required additional medical intervention. [2]

### Statistical analysis

Categorical variables were presented as numbers (%) and compared by Chi-square test or Fisher’s exact test for univariate analysis. We analyzed the normality of continuous variables by Shapiro–Wilk normality test, and normal distribution was not universally noted. Thus, continuous variables were described by median (interquartile range [IQR]) and compared by Mann–Whitney U test for univariate analysis. Odds ratio (OR) and corresponding confidence intervals of 95% (95%CI) were used to estimate the association of variables and outcomes. In the multivariable analysis, binary logistic regression model with method of Backward LR was used to identify the independent predictive factors of acute CVEs in AECOPD patients. Variables with *P* < 0.001 in the univariate analysis were included into the multivariable analysis.

From the conservative point of view, we managed missing data with the following procedure: firstly, patients with many missing variables were excluded; secondly, variables with missing number ≥ 5% were removed; thirdly, variables with missing number < 5% were supplemented with the negative value. Statistical analysis was performed using IBM SPSS statistics 23 (SPSS Inc, Chicago, IL), and statistical graph was generated with GraphPad Prism 6 (GraphPad Software, CA, USA). The statistical significance level was set as a two-tailed *P* < 0.05.

## Result

### Baseline characteristics

**Fig. 1:**
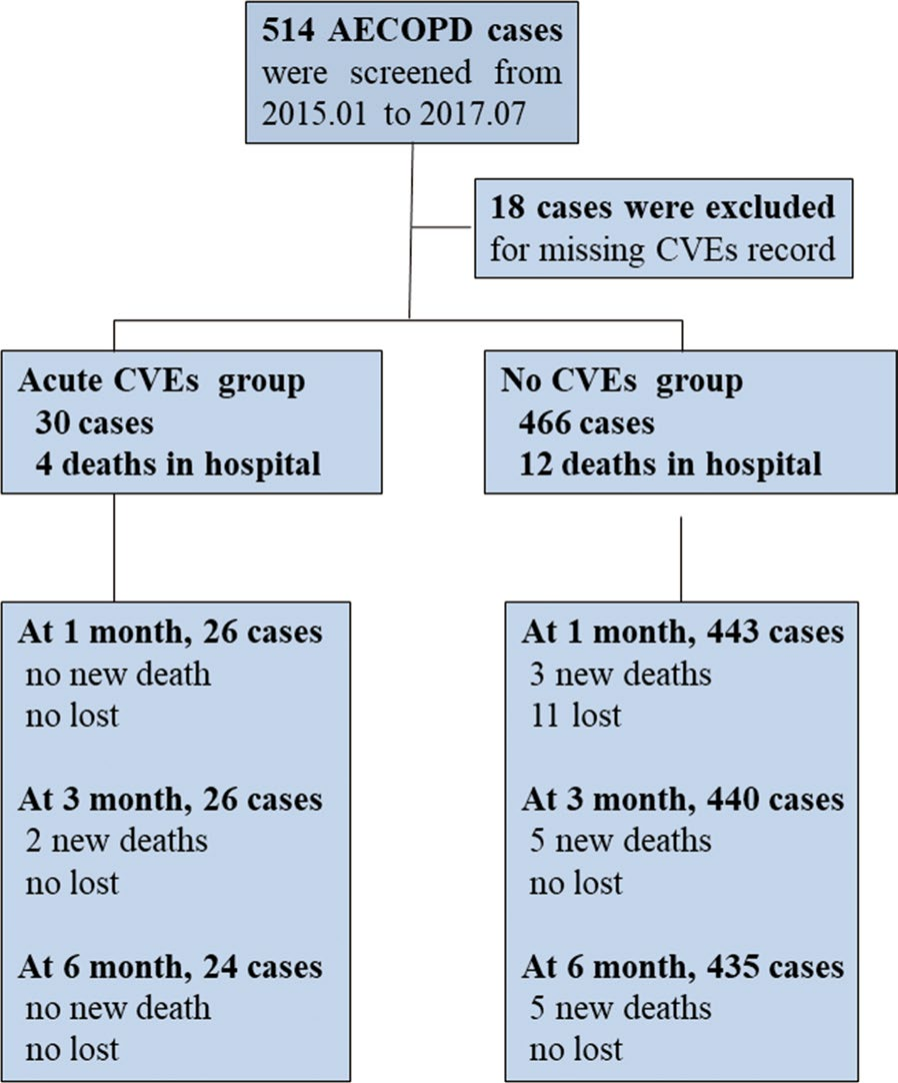
Flow chart of the study. AECOPD, acute exacerbation of chronic obstructive pulmonary disease; CVEs, cardiovascular events

Between January, 2015 and July, 2017, 514 AECOPD cases were collected in the two hospitals. After excluding those without any record of acute CVEs during hospitalization, we included 496 cases into analysis. (Fig. 1) A total of 30 cases (6.05%) had concomitant acute CVEs (ACS, n = 2; arrhythmia, n = 19; LVD, n = 13), and 4 cases died in hospital. At 1 month after discharge, 11 patients were lost to follow up.

As shown in Table 1, patients from the acute CVEs group were older than those from the non-CVEs group (*P* = 0.027). Interestingly, females were more susceptible to have acute CVEs during the AECOPD period (*P* = 0.037, OR = 2.35, 95% CI = 1.03–5.33). As for severity of COPD, two group did not significantly differ in the grading by spirometric values, symptom scores and number of previous 1-year AE.

**Table 1:** Baseline characteristics and cardiovascular risk factors of study population

Category	Measurements	Acute CVEs group (n = 30)	No CVEs group (n = 466)	*P* value
Basic information	Age (year)	82 (77–84)	78 (68–84)	0.025
	Male	21 (70.0%)	394 (84.5%)	0.037
	BMI	22.8 (20.0–24.2) (n = 15)	21.7 (20.2–25.4) (n = 314)	NS
	Smoking history (yes or no)	21 (70.0%)	378 (81.1%)	NS
	Smoking history (pack*year)	43.5 (31.8–57.5) (n = 12)	46.0 (34.3–60.0) (n = 192)	NS
COPD severity	FEV_1_ (% predicted)	40.2 (29.3–53.7) (n = 15)	37.2 (28.4–49.0) (n = 300)	NS
	GOLD grade I	0 (0%)	1 (0.3%)	NS
	GOLD grade II	2 (13.3%)	66 (22.0%)	
	GOLD grade III	6 (40.0%)	144 (48.0%)	
	GOLD grade IV	5 (33.3%)	89 (29.7%)	
	mMRC score	3 (2–3) (n = 17)	3 (2–3) (n = 324)	NS
	CAT score	23.5 (12.3–26.8) (n = 16)	22.0 (15.0–28.0) (n = 319)	NS
Cardiovascular risk factors	Hypertension	7/22 (31.8%)	169/380 (44.5%)	NS
	Diabetes	6/22 (27.3%)	50/380 (13.2%)	0.063
	Hyperlipemia^a^	0/16 (0%)	14/333 (4.2%)	NS
	Coronary heart disease	10/22 (45.5%)	86/377 (22.8%)	0.016
	Cerebrovascular disease	2/21 (9.5%)	18/363 (5.0%)	NS
	Left heart insufficiency	8/22 (36.4%)	31/379 (8.2%)	< 0.001
Other respiratory diseases	Asthma	1/22 (4.5%)	3/377 (0.8%)	NS
	Bronchiectasis	5/22 (22.7%)	60/379 (15.8%)	NS
	Interstitial lung changes	2/19 (10.5%)	26/341 (7.6%)	NS
	Lung cancer	0/22 (0%)	8/379 (2.1%)	NS
	Ex-tuberculosis	2/22 (9.1%)	94/379 (24.8%)	NS
COPD-related treatment in stable period	Regular LAMA	6/24 (25.0%)	137/390 (35.1%)	NS
	Regular ICS/LABA	5/24 (20.8%)	170/388 (43.8%)	0.027
	Regular ICS/LABA/LAMA	2/24 (8.3%)	94/387 (24.3%)	NS
	Regular theophylline	5/24 (20.8%)	97/384 (25.3%)	NS
	Oxygen therapy	10/24 (41.7%)	167/388 (43.0%)	NS
	Noninvasive mechanical ventilation	3/23 (13.0%)	40/372 (10.8%)	NS
Pre-study exacerbations in 12 months	Total numbers	2 (1–2.5) (n = 17)	2 (1–3) (n = 337)	NS
	Hospitalization numbers	2 (1–2) (n = 17)	1 (1–2) (n = 335)	0.025

Data were shown as number (percentage), n/N (percentage) or median (IQR). *P*-values between two group were calculated by Fisher’s exact test, Chi-square test, or Mann–Whitney U test

*CVEs* cardiovascular events, *BMI* body mass index, *FEV*_*1*_ forced expiratory volume in one second, *COPD* chronic obstructive pulmonary disease, *GOLD* Global Initiative for Chronic Obstructive Lung Disease, *mMRC* modified Medical Research Council, *CAT* COPD Assessment test, *ICS* inhaled glucocorticoid, *LABA* long-acting beta agonist, *LAMA* long-acting muscarinic antagonist

^a^Hyperlipemia was defined as total cholesterol > 5.2 mmol/L in the AE period, for lack of lipid profile examination in the stable period

The presence of other coexisting respiratory diseases were not associated with acute CVEs. On the other hand, prior cardiovascular diseases were strong predictors of acute CVEs in AECOPD: coronary heart disease (*P* = 0.016, OR = 2.82, 95% CI = 1.18–6.75) and left heart insufficiency (*P* < 0.001, OR = 6.42, 95% CI = 2.50–16.48). When defined as total cholesterol > 5.2 mmol/l, hyperlipidemia was not associated with increased risk of acute CVEs. Regular usage of inhaled agents for treating COPD in the stable period was a protective factor, especially when inhaled corticosteroid (ICS) and long-acting beta agonist (LABA) combination was used (*P* = 0.027, OR = 0.34, 95% CI = 0.12–0.92).

### Acute CVEs were associated with poor outcomes in AECOPD patients

In comparison with non-CVEs group, increased death risk in hospital was observed in the acute CVEs group, with the constituent ratio of 13.3% versus 2.6% (*P* = 0.001, OR = 5.81, 95% CI = 1.75–19.26). (Table 2) Moreover, patients with acute CVEs had longer hospital stay and more frequent re-exacerbation of COPD within a 3 month period.

**Table 2:** Clinical outcomes between acute CVEs group and normal group

Outcomes	Acute CVEs group (n = 30)	No CVEs group (n = 466)	*P* value	Odds ratio	95% CI
Hospital mortality	4/30 (13.3%)	12/466 (2.6%)	0.001	5.821	1.75–19.30
ICU admission	2/30 (6.7%)	26/466 (5.6%)	NS		
Hospital LOS (d)	14 (12–18)	13 (10–15)	0.022		
Re-AE at 1 month	8/26 (30.8%)	64/443 (14.5%)	0.025	2.632	1.10–6.31
Accumulative death at 1 month	4/30 (13.3%)	15/455 (3.3%)	0.006	4.513	1.40–14.57
Re-AE at 3 month	14/26 (53.8%)	138/440 (31.4%)	0.018	2.553	1.15–5.67
Accumulative death at 3 month	6/30 (20%)	20/455 (4.4%)	< 0.001	5.438	1.99–14.79
Re-AE at 6 month	15/24 (62.5%)	195/435 (44.8%)	NS		
Accumulative death at 6 month	6/30 (20%)	25/455 (5.5%)	0.002	4.30	1.61–11.47

Data were shown as n/N (percentage) or median (IQR). *P*-values between two group were calculated by Fisher’s exact test, Chi-square test, or Mann–Whitney U test

*CVEs* cardiovascular events, *CI* confidence index, *ICU* intensive care unit, *LOS* length of stay, *AE* acute exacerbation

### Risk factors of acute CVEs identified by univariate analysis

As shown in Table 3, common respiratory symptoms such as cough, expectoration, hemoptysis, shortness of breath and chest pain, were not indicators of acute CVEs,. Additionally, fever, chill and cyanosis were not related to acute CVEs. Abnormal increase in heart rate and new or worsening edema of both lower limbs indicated subsequent cardiac deterioration.

**Table 3:** Other cardiovascular risk factors in the univariate analysis

Category	Variables	Acute CVEs group (n = 30)	No CVEs group (n = 466)	*P* value	Odd ratio	95% CI
Symptoms and signs	Palpitation	2/20 (10.0%)	9/388 (2.3%)	0.039	4.68	0.94–23.27
	20% increase in HR	5/20 (25.0%)	11/386 (2.8%)	< 0.001	11.36	3.50–36.86
	Both lower limb edema	10/20 (50.0%)	70/389 (18.0%)	< 0.001	4.56	1.83–11.37
	Disturbance of consciousness	4/20 (20.0%)	19/387 (4.9%)	0.004	4.84	1.48–15.90
Laboratory tests	Neutrophil (%)	80.85 (73.18–86.05)	75.50 (67.55–83.50)	0.035		
	≥ 80	16/30 (53.3%)	165/466 (35.4%)	0.048	2.09	0.99–4.38
	CRP (mg/L) (n = 478)	33.4 (12.45–68.55)	19.2 (4.95–61.1)	NS		
	≥ 10	24/29 (82.8%)	280/449 (62.4%)	0.028	2.90	1.09–7.74
	Urea nitrogen (mmol/L)	6.80 (4.55–9.88)	5.50 (4.30–7.10)	0**.**031		
	≥ 7.5	14/30 (46.7%)	102/459 (22.2%)	0.002	3.06	1.45–6.49
	cTnT (ng/mL) (n = 333)	0.02 (0.01–0.03)	0.01 (0–0.02)	0.054		
	> 0.03	6/28 (21.4%)	39/304 (12.8%)	NS		
	LDH (U/L)	316 (198–404)	208 (181–257)	0.002		
	> 245	16/25 (64.0%)	120/413 (29.1%)	< 0.001	4.34	1.87–10.10
	NT-proBNP (pg/ml) (n = 218)	993 (268–1875)	296 (139–1070)	0.021		
	≥ 300	13/17 (76.5%)	100/201 (49.8%)	0.043		
	D-dimer (ug/L)	0.77 (0.43–1.21)	0.52 (0.27–1.04)	NS		
	≥ 0.5	16/25 (64.0%)	235/448 (52.5%)	NS		
	FBG (mmol/L)	7.0 (5.6–7.3)	5.5 (4.7–6.8)	0.007		
	≥ 7	9/27 (33.3%)	95/421 (22.6%)	NS		
	Cholesterol (mmol/L)	3.90 (2.92–4.34)	4.19 (3.61–4.98)	0.016		
	LDL (mmol/L)	2.20 (1.58–2.67)	2.62 (2.11–3.11)	0.013		
	PaO2 (mmHg)	80.50 (64.25–108.25)	78.5 (65–100)	NS		
	≤ 60	4/28 (14.3%)	80/422 (19.0%)			
	PaCO2 (mmHg)	51.0 (39.0–66.0)	47 (41–58)	NS		
	≥ 50	15/27 (55.6%)	185/416 (44.5%)			
CT manifestation	Pleural effusion	9/25 (36.6%)	75/389 (19.3%)	0.046	2.34	1.00–5.50
	multiple lobes' lesion	6/23 (26.1%)	94/390 (24.1%)	NS		
Complications	Pneumothorax	4/30 (13.3%)	1/466 (0.2%)	< 0.001	71.54	7.71–663.4
	Pulmonary embolism	2/30 (6.7%)	3/457 (0.6%)	0.03	10.81	1.73–67.38
	Electrolyte disturbance	11/30 (36.7%)	53/462 (11.5%)	< 0.001	4.47	2.02–9.90

Data were shown as n/N (percentage) or median (IQR). *P*-values between two group were calculated by Fisher’s exact test, Chi-square test, or Mann–Whitney U test

*CVEs* cardiovascular events, *CI* confidence index, *HR* heart rate, *CRP* C-reactive protein, *ESR* erythrocyte sedimentation rate, *cTnT* cardiac muscle isoform of troponin T, *LDH* lactate dehydrogenase, *NT-pro BNP* N-terminal pro-brain natriuretic peptide, *FBG* fasting blood glucose, *LDL* low-density lipoprotein, *PaO*_*2*_ partial pressure of oxide in artery, *PaCO*_*2*_ partial pressure of carbon dioxide in artery, *CT* computed tomography

Elevated neutrophils and C-reactive protein, suggesting aggravated inflammation, had a weak association with acute CVEs. Procalcitonin and erythrocyte sedimentation rate were excluded from the analysis, because they were not routinely tested for in patients with AECOPD. Indices of myocardial damage and heart failure, namely lactic dehydrogenase and N-terminal proB-type natriuretic peptide (NT-proBNP), were significantly up-regulated in the acute CVEs group. As a promising variable in COPD management, neither absolute counts nor binary classification (150/µl) of eosinophils had any statistical association with acute CVEs. Additionally, patients in the acute CVEs groups had more complications in the AE period, including pneumothorax, pulmonary embolism and electrolyte disturbance. Whereas, pneumonia and respiratory failure were not associated with acute CVEs.

**Fig. 2:**
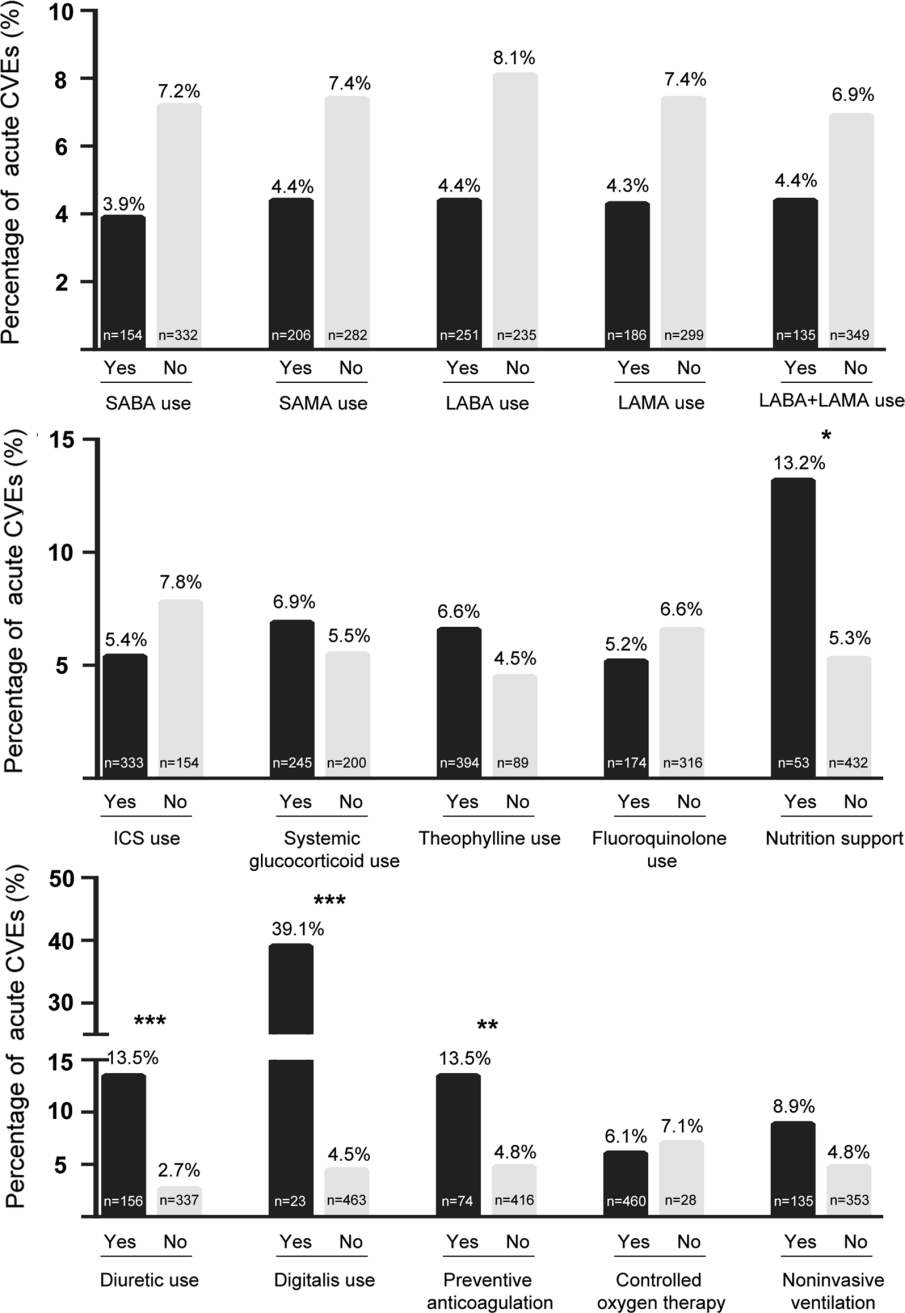
Commonly-used medications and acute CVEs. Usage of SABA, SAMA, ICS here included either aerosol inhalation or specialized commercial agents. CVEs, cardiovascular events; SABA, short-acting beta agonist; SAMA, short-acting muscarinic antagonist; LABA, long-acting beta agonist; LAMA, long-acting muscarinic antagonist; ICS, inhaled glucocorticoid. *P < 0.05; **P < 0.01; ***P < 0.001

As indicated in Fig. 2, use of inhaled beta receptor agonists and muscarinic agonists in the AE period was not associated with an increased occurrence of acute CVEs but had slightly protective effects. Interestingly, use of inhaled glucocorticoid agents had a tendency to prevent acute CVEs (*P* = 0.066, OR = 0.49, 95% CI = 0.22–1.02), compared with aerosol inhalation of venous glucocorticoid agents (*P* = 0.22). Among 496 cases, 490 cases received antibiotics, of whom nearly 1/5 received combined antibiotic therapy. Although use of fluoroquinolone was found to be risk factor for QTc prolongation in a previous study, [15] it was not statistically associated with acute CVEs in our cohort. In addition, 3 cases received macrolides and 6 cases received anti-fungal agents, and were thus not included into statistical analysis.

Preventive anticoagulation and nutrition support were predictors of acute CVEs, which might be attributed to poor baseline status of patients. (Fig. 2) Nine of 23 patients using digitalis had acute CVEs in the AE period, with 3 being treated for LVD and another 6 for controlling ventricular rate.

### Increased heart rate, electrolyte disturbance and use of diuretics were independent risk factors

After removing 90 cases with missing data, 406 cases (20 CVEs and 386 non-CVEs) were included into the multivariate analysis. In Table 4, a total of eight variables with *P* < 0.001 in the univariate analysis were included into the binary logistic regression equation. Previous LVD, 20% increase in heart rate (HR), electrolyte disturbance and diuretics use were independent predictors of acute CVEs in the AE period. Moreover, approximately 1/3 of the patients receiving diuretics had electrolyte disturbance at admission.

**Table 4:** Predictors of acute CVEs in a multivariable logistic regression model

Characteristic	Adjusted OR	95% CI	Adjusted *P*-value
Previous left heart insufficiency	5.06	1.66–15.36	0.004
20% increase in HR	10.19	2.21–46.99	0.003
Both lower limb edema			NS
LDH ≥ 245 U/L			NS
Electrolyte disturbance	4.24	1.40–12.77	0.01
Pneumothorax			NS
Diuretics use	6.37	1.96–20.67	0.002
Digitalis use			NS

*CVEs* cardiovascular events, *OR* odds ratio, *CI* confidence index, *HR* heart rate, *LDH* lactic dehydrogenase, *NS* non-significance

## Discussion

We demonstrated that the development of acute CVEs during AECOPD period was not only associated with increased hospital mortality but also with increased risks of short-term re-AE after discharge. Moreover, previous LVD, increased HR, electrolyte disturbance and diuretics use were identified as independent risk predictors of CVEs.

In line with our findings of increased HR and acute CVEs, other investigators also noted that increased resting HR was associated with a shortened life expectancy and increased cardiovascular mortality in COPD patients irrespective of the spirometric grading [21]. However, the relationship between electrolyte imbalance and CVEs remains to be confirmed. Our findings partly validated previous results of poor outcomes of hyperphosphatemia (only for male) [22] and hypocalcemia [23] in AECOPD patients. Whereas, no statistical difference in sodium, potassium and chlorides levels was observed between acute CVEs and non-CVEs group in our cohort. Another study also reported no correlation between electrolyte imbalance and QTc prolongation in hospitalized patients with COPD [24]. As for diuretics use, a retrospective study reported that prescription of loop diuretics increased the risks of AE and death in the elderly patients with COPD [25]. In our cohort, we defined the diuretic usage as using loop diuretics and spironolactone, and found that diuretic usage was independently associated with acute CVEs. Contrarily, thiazide diuretics were recommended as the first-line antihypertensive agents for COPD patients, since it did not cause an increase in the numbers of AE [26]. Thus, mild diuretics and low-dosage might be more suitable for patients with AECOPD.

We speculate that cardiovascular risks of diuretics might result from electrolyte imbalance, hypovolemia or pre-existing heart failure. Moreover, increased HR is also one of the signs of hypovolemia. Two typical cases of acute CVEs in our cohort demonstrated that inappropriate usage of diuretics could result in electrolyte imbalance or hypovolemia. Patient A was a typical case, whose electrolyte disturbance led to worsening of his previous LVD. Before admission, he had edema of both lower extremities and took oral diuretics on his own. On day 1 in hospitalization, he suddenly complained of dyspnea and orthopnea. Immediate laboratory tests showed he had hypokalemia (2.6 mmol/L), and hypochloridemia (96 mmol/L). Then, he was given potassium supplementation, digitalis, amrinone, intravenous and oral diuretics, along with noninvasive mechanical ventilation. On day 15, he recovered and was discharged from hospital. Patient B was another typical example, whose hypovolemia led to the occurrence of arrhythmia. He had no edema of both lower extremities before admission. He was prescribed with intravenous diuretics on day 1–4 and converted into oral diuretics on day 5–7. On day 7 of hospitalization, he suddenly presented with a newly onset of atrial fibrillation. Immediate laboratory tests showed he had hypovolemia (erythrocyte counts = 5.88*10^12^ and hemoglobin = 184 g/L). He was given appropriate fluid infusion to expand blood volume, and discontinued diuretics. He was also given propafenone for cardioversion and oral amiodarone for maintenance. On day 9, sinus rhythm was restored and patient was discharged from hospital on day 12. Thus, the above two cases suggested that electrolytes and blood volume should be closely monitored during the AE period, especially in the patients receiving diuretics. As a golden standard of hemodynamic assessment, right heart catheterization is, however, limitedly carried out in patients with AECOPD due to its invasiveness and patients’ poor status. Hence, new echocardiographic parameters are recommended in the clinical setting, including tricuspid annular plane systolic excursion (TAPSE) and systolic S′ velocity of the tricuspid annulus. [27]

Similar to our results of increased NT-proBNP in acute CVEs group, NT-proBNP levels [28] were previously found to be strong indicators of death in patients with AECOPD. Likewise, Smith GL reported that increased urea nitrogen was associated with cardiovascular mortality in the elderly. [29] Although Smith GL showed the association of increased creatinine level with myocardial infarction (> 88.4 mmol/L) and heart failure (> 97.2 mmol/L) [29], we did not confirm this association in our study. Theophylline, fluoroquinolone and inhaled bronchodilators in AE period, which were previously regarded as cardiovascular risk factors [14–16], were not statistically associated with acute CVEs in our cohort.

Since nearly half of AEs are caused by lower respiratory bacterial infection [30], we tried to gain insights from some studies of Community-Acquired Pneumonia (CAP) and associated cardiac complications. Similar to our study, two observational studies reported that incident CVEs was a strong negative indicator of 30-day survival of patients with CAP [31, 32]. In the comprehensive analysis of three CAP studies (25–40% of subjects with chronic respiratory diseases), several risk factors for acute CVEs including age, preexisting coronary heart disease, diabetes, congestive heart failure, pleural effusion, increased pulse, urea nitrogen, and blood glucose had been identified [31–33]. These finding are consistent with our results.

Some limitations should be noted before interpreting this study. As a nested case–control study, absolute causal relationship cannot be confirmed, and incidence and mortality of acute CVEs in patients with AECOPD cannot be calculated. Second, due to a limited number of acute CVEs and statistical efficacy, some risk factors might have been missed. We plan to further validate our results in a large-scale and multicenter cohort with more outcomes of CVEs. Third, some baseline information in the stable period was missing, and use of cardioprotective medications e.g. anti-platelet agents and statins was not fully recorded,, which might result in biases [14, 34]. Fourth, baseline comorbidities were reported by patients themselves and not validated by detailed laboratory tests, which might have led to recall bias. Fifth, we did not take some useful scales of cardiovascular risk assessment into consideration [35].

## Conclusion

Cardiac complications in AE period were significantly associated with poor outcomes in patients with COPD. Patients with previous history of LVD and 20% increase in HR were susceptible to cardiac complications and needed close monitoring. Furthermore, diuretics use in AE period might be associated with underlying cardiovascular risks, and electrolytes and blood volume should be carefully assessed.

## Data Availability

The datasets used and/or analyzed during the current study available from the corresponding author on reasonable request.

## Abbreviations

COPD: Chronic obstructive pulmonary disease
CVEs: Cardiovascular events
AE: Acute exacerbation
ACS: Acute coronary syndrome
LVD: Left ventricular disfunction
HR: Heart rate
CVD: Cardiovascular disease
mMRC: Modified Medical Research Council
CAT: COPD assessment test
GOLD: Global Strategy for Prevention, Diagnosis and Management of COPD
re-AE: Recurrent acute exacerbation
NT-proBNP: N-terminal proB-type natriuretic peptide
SpO2: Oxygen saturation
cTnI: Cardiac troponin I
RHF: Right heart failure
TAPSE: Tricuspid annular plane systolic excursion
CAP: Community-acquired pneumonia
